# Enhanced Performance and Mode of Action of a Novel Antibiofilm Hydrofiber® Wound Dressing

**DOI:** 10.1155/2016/7616471

**Published:** 2016-11-20

**Authors:** David Parsons, Kate Meredith, Victoria J. Rowlands, Darryl Short, Daniel G. Metcalf, Philip G. Bowler

**Affiliations:** ^1^Science & Technology, ConvaTec GDC, First Avenue, Deeside Industrial Park, Flintshire CH5 2NU, UK; ^2^Microbiology Services, Research & Development, ConvaTec GDC, First Avenue, Deeside Industrial Park, Flintshire CH5 2NU, UK; ^3^Analytical Services, Research & Development, ConvaTec GDC, First Avenue, Deeside Industrial Park, Flintshire CH5 2NU, UK

## Abstract

Biofilm development in wounds is now acknowledged to be a precursor to infection and a cause of delayed healing. A next-generation antibiofilm carboxymethylcellulose silver-containing wound dressing (NGAD) has been developed to disrupt and kill biofilm microorganisms. This* in vitro* study aimed to compare its effectiveness against various existing wound dressings and examine its mode of action. A number of biofilm models of increasing complexity were used to culture biofilms of wound-relevant pathogens, before exposure to test dressings. Confocal microscopy, staining, and imaging of biofilm constituents, total viable counting, and elemental analysis were conducted to assess dressing antibiofilm performance. Live/dead staining and viable counting of biofilms demonstrated that the NGAD was more effective at killing biofilm bacteria than two other standard silver dressings. Staining of biofilm polysaccharides showed that the NGAD was also more effective at reducing this protective biofilm component than standard silver dressings, and image analyses confirmed the superior biofilm killing and removal performance of the NGAD. The biofilm-disruptive and silver-enhancing modes of action of the NGAD were supported by significant differences (*p* < 0.05) in biofilm elemental markers and silver donation. This* in vitro* study improves our understanding of how antibiofilm dressing technology can be effective against the challenge of biofilm.

## 1. Introduction

Antibiotics and topical antiseptics are commonly used in wound care to control wound microbial bioburden and hence facilitate healing. In order for any antibiotic or antiseptic to be effective, it needs to directly contact the microbial cell in order to induce static or “cidal” effects. However, local factors within a wound environment often impede the effectiveness of such antimicrobial agents. If a wound is poorly perfused and is harbouring antibiotic-resistant microorganisms, then the effectiveness of a systemically administered antibiotic is likely to be uncertain [1]. If an antiseptic is delivered via a wound dressing, then the dressing must be able to make the antiseptic available to microbial cells; otherwise its effectiveness will be suboptimal [2]. The variability in the availability of silver from a variety of wound dressings has previously been demonstrated* in vitro *[2].

Another potential barrier to both antiseptics and antibiotics in wounds is biofilm. Biofilm is a self-expressed extracellular matrix produced by microorganisms that protects them from environmental hostilities such as antimicrobial agents and immune cells [3]. The prevalence of biofilm in nonhealing wounds is increasingly recognised [4, 5], and the persistence and recurrence of infections are most likely attributed to the biofilm effect and consequent tolerance to antimicrobial agents [6]. With this in mind, there is a clear clinical need to facilitate antimicrobial effectiveness (both antibiotics and antiseptics) by introducing antibiofilm substances that are able to break down biofilm in wounds and expose associated microorganisms to antimicrobial attack.

A next-generation antimicrobial Hydrofiber dressing (NGAD; AQUACEL® Ag+ Extra™) has recently been developed and was designed to disperse wound biofilm and enhance the antimicrobial action of ionic silver [7]. In this study,* in vitro* biofilm models and microscopic, microbiological, and analytical chemistry methods were developed to examine the effectiveness of the NGAD at killing biofilm-associated bacteria, including antibiotic-resistant bacteria, and its ability to remove dispersed biofilm compared to standard antimicrobial dressings. Further, this work also aimed to investigate the mode of action of the NGAD and the ability of this antibiofilm dressing to disrupt biofilm and enhance silver penetration into biofilm.

## 2. Materials and Methods

### 2.1. Biofilm Preparation

**Figure 1:**
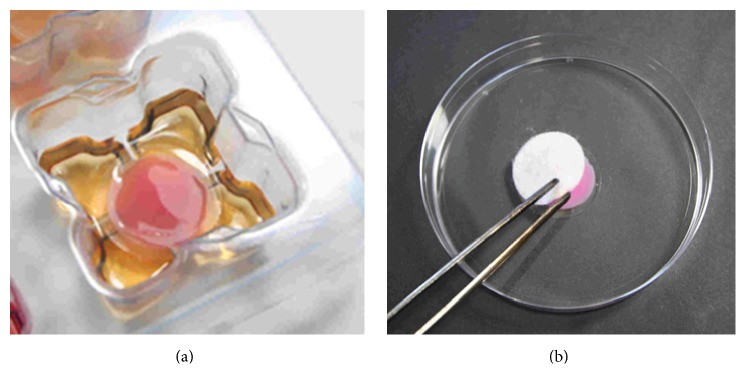
(a) 24-hour* S. aureus* biofilm supported on a 25 mm filter disc in contact with BGM in a deep 6-well plate (biofilm is stained pink for clarity). (b) Test dressing application to biofilm (biofilm is stained pink for clarity).

Individual strains of challenge microorganisms (Table 1) were grown to log-phase in Tryptone Soy Broth (TSB) and then diluted with the appropriate biofilm growth medium (BGM, Table 1) to approximately 1 × 10^5^ cfu/mL. 7 mL aliquots of BGM were dispensed into each well of deep 6-well plates (BD Biosciences). Anodisc filters (25 mm dia., 0.2 *μ*m pore size; Whatman) were carefully placed onto the support ribs within each well such that the BGM was only in contact with the downward-facing surface. Aliquots of microbial suspensions (0.1 mL aliquots of 1 × 10^5^ cfu/mL suspension for single-species models; 45 *μ*L of* S. aureus* and 5 *μ*L of* K. pneumoniae* 1 × 10^5^ cfu/mL suspensions for the polymicrobial model, Table 1) were pipetted onto the centre of the upper surface of each filter disc. The plate lid was replaced and the plate incubated at 35 ± 3°C. After 24 hours (Figure 1(a)) filter discs were removed and rinsed by moving the filter backwards and forwards 10 times with forceps in 30 mL of 0.85% w/v saline to remove planktonic microorganisms and unattached matter. The filter disc-supported biofilms were then used immediately in either a simple biofilm model or a simulated wound polymicrobial biofilm model, to test various dressings and analyse their effects using multiple methodologies (Table 1).

### 2.2. Dressing Applications

Dressings tested are described in Table 2. Note that for analysis of K^+^, Mg^2+^, Ca^2+^, and Zn^2+^ ions in residual biofilm it was only possible to reliably test the CMC-containing dressings, which have a proprietary elemental composition known to the authors. The K^+^, Mg^2+^, Ca^2+^, and Zn^2+^ contents of the NCSC and SNAD dressings are unknown.

### 2.3. Simple Biofilm Model

Filter disc-supported biofilms were placed biofilm uppermost into individual 55 mm Petri dishes. 24 mm diameter circles of the test dressings were applied (Figure 1(b)) as stated in the respective manufacturer's instructions for use, hydrating with sterile water or saline as indicated (e.g., for CMC, SCMC, and the NGAD, this was 0.7 mL aliquots of sterile saline). Dressings were left in contact with the biofilm for 24 or 48 hours (Table 1) at 35 ± 3°C in the closed Petri dishes, following which the dressings were gently removed by gripping one edge with forceps and rolling back the dressing. The exposed residual biofilm-containing filter disc was analysed immediately. A minimum of six replicates were performed for each test dressing and no-dressing control.

### 2.4. Simulated Wound Polymicrobial Biofilm Model

**Figure 2:**
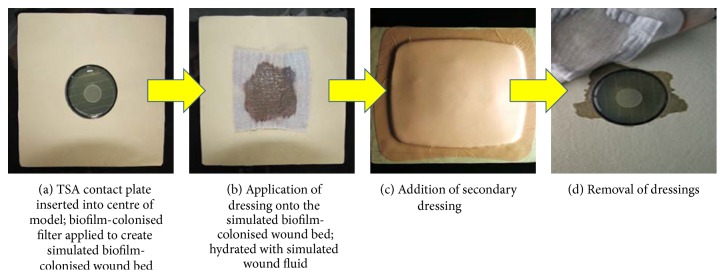
Simulated wound polymicrobial biofilm model with the NGAD and AQUACEL Foam secondary dressing application within the wound assembly.

In a more complex model, simulated wound set-ups were created by covering Perspex plates with bovine leather (simulating periwound skin) and cutting out a circular hole into which a 55 mm Tryptone Soy Agar (TSA) contact plate (simulating a moist wound bed containing a reservoir of isotonic nutrients) could be tightly fitted [7, 9]. 24-hour, filter disc-supported,* S. aureus-K. pneumoniae* biofilms were centrally placed on the plate, biofilm uppermost (Figure 2(a)). Test dressings were applied and hydrated with the relevant amount of simulated wound fluid (50% foetal calf serum and 50% maximal recovery diluent) using manufacturer's instructions as a guide (Figure 2(b)). The hydrated test dressing was then covered with an appropriate secondary dressing (AQUACEL Foam dressing) (Figure 2(c)). The assembled test models were incubated for 48 hours at 35 ± 3°C before the dressings were removed as in the simple biofilm model (Figure 2(d)).

### 2.5. Biofilm Analyses

#### 2.5.1. Staining

A staining procedure was undertaken to determine the viability of biofilm bacteria prior to and after exposure to the test dressings. Using both the simple biofilm and the simulated wound polymicrobial biofilm models, biofilm controls (at the start and end of each experiment) and residual biofilm samples (after dressing removal) were exposed to* Live/Dead® BacLight* stain (Molecular Probes, Invitrogen) for 10 minutes at room temperature in darkness prior to analysis. Calcofluor White (Fluka® Analytical) staining at room temperature in darkness was used to establish the effect of the dressings on the biofilm extracellular polymeric substances (EPS). In the case of the simulated wound polymicrobial biofilm model, peptide nucleic acid fluorescence* in situ* hybridisation (PNA FISH, AdvanDx Inc.) was used to differentiate the two organisms (*S. aureus* and* K. pneumoniae*) using fluorescent labels to enable visualisation of the polymicrobial biofilm population. PNA FISH was conducted according to the manufacturer's instructions, with the exception of a 90-minute hybridisation step at 55 ± 1°C, and omission of a water rinse step. Stained samples were examined immediately using confocal laser scanning microscopy (CLSM; Leica TCS SP2, Leica Microsystems) and images were captured for later analysis.

#### 2.5.2. Image Analysis

Image analysis was undertaken to determine the viability of bacteria throughout the depth of each biofilm of antibiotic-resistant* P. aeruginosa* from the simple biofilm model after exposure to silver dressings. The CLSM uses a highly focused laser beam to illuminate the test sample at right-angles to the direction of observation. The width of the beam is very narrow and its position (distance from the observer) can be closely controlled; therefore thin layers at different depths within the sample can be observed sequentially. By using selective stains which have specific emission wavelengths (colours) and coloured filters, individual components of the sample can be differentiated. Images (each approximately 100 × 100 *μ*m in area) were captured at intervals of 0.5 *μ*m vertically apart throughout the full thickness (depth) of the residual biofilm, down to the supporting filter. Qualitative and semiquantitative data were obtained by recombining each series of layers to provide a three-dimension reconstruction of the sample using* Image-Pro Premier *3D software. Quantitative image analysis was performed on live/dead stain images of a selected range of dressings using* Image-Pro Plus*® version 7.0 software. For each image the number of objects (cells) of area greater than 10 pixels (determined by observation to be the approximate minimum size of an individual bacterium) was counted for the green (live) and red (dead) coloured series. A minimum threshold of 10 counted items per layer was set as the criteria for the confirmed presence of biofilm and to be valid for use in any further calculation. Objects that were much greater in area than bacterial cells and were clearly not of bacterial origin were excluded. Three separately treated biofilms were analysed for each of the selected dressings, observing each biofilm at multiple sites across the sample. The only exclusion criteria were if image quality was inadequate (i.e., poor focus or interfering image artifact) or if there was an incomplete data set (i.e., no upper and/or lower boundary of the biofilm could be identified).

#### 2.5.3. Quantitative Microbiology

In the case of the simulated wound polymicrobial biofilm model, total viable counts were performed in triplicate on the polymicrobial biofilms to allow comparison with PNA FISH microscopy data.

#### 2.5.4. Elemental Analysis

After 24 or 48 hours of incubation with the test dressings, the filter disc-supported single-species biofilms were placed separately into individual plastic sample tubes containing 10 mL of 1.2 M aqueous hydrochloric acid. Tubes were agitated for 10 minutes or until all of the residual biofilm had visibly dissolved. The resultant solutions were filtered through 0.45 *μ*m membrane filters (Whatman) to remove any bacteria or dressing fibres and then assayed for solubilized potassium (K^+^), magnesium (Mg^2+^), calcium (Ca^2+^), zinc (Zn^2+^), and silver (Ag^+^) ions by inductively coupled plasma mass spectrometry (ICP-MS, Agilent Technologies 7700 Series).

#### 2.5.5. Statistical Analysis

Student's 2-sample *t*-tests and one-way analysis of variance (ANOVA) (Minitab® software) were performed, where possible, to determine any statistically significant differences (*p* < 0.05) between dressing performances.

## 3. Results

### 3.1. Biofilm Models

Table 3 illustrates the reproducibility of the various biofilm models used in this* in vitro* study. Irrespective of the model, bacterial strain, duration, analytical method, or sample size, the relatively low standard deviation of the data from different biofilm characteristics indicates good reproducibility.

### 3.2. Dressing Effectiveness

**Figure 3:**
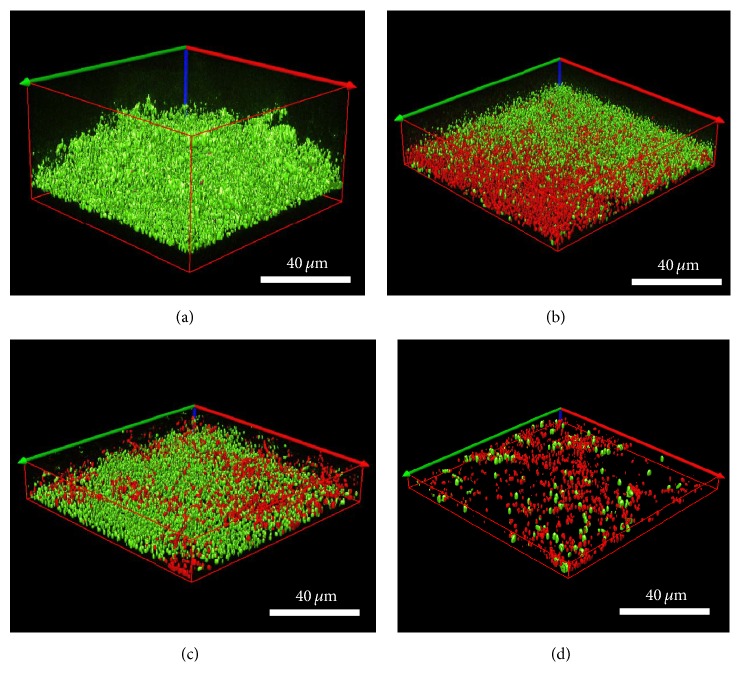
Representative isosurface 3D imaging, performed using the* Image-Pro Premier® *3D software of the CLSM images biofilms stained with* BacLight*® (green = viable bacteria; red = nonviable bacteria). (a) Initial biofilm *T*_24 hours_. (b) NCSD after 48 hours of exposure. (c) SNAD after 48 hours of exposure. (d) NGAD after 48 hours of exposure.

*Live/Dead BacLight* selectively stains bacteria (viable cells appear green whereas nonviable (dead) cells appear red). Confocal images demonstrated that all three silver test dressings significantly reduced polymicrobial biofilm thickness (*μ*m) compared to the control (initial biofilm *T*_24 hours_) (*p* = 0.000 for all dressings) (Table 4). Following ANOVA, simultaneous confidence interval comparisons demonstrated that there were statistically significant differences between silver dressings. The NGAD resulted in significantly thinner residual biofilms than NCSD and SNAD (*p* = 0.000 in both instances), while there was no significant difference in biofilm thickness reduction between NCSD and SNAD exposure (*p* = 0.065). Confocal images also illustrate these differences in biofilm thickness (Figures 3(b)–3(d)) and that there were consistently more dead cells under the NGAD (Figure 3(d)) and less cells overall, compared to the other two dressings (Figures 3(b) and 3(c)). Following dressing application, the volume of green Syto®9 stain (indicative of remaining viable biofilm bacteria) was 7.8% beneath the NGAD, compared to 51.5% under NCSD and 44.1% under SNAD, for the images shown in Figures 3(b)–3(d).

#### 3.2.1. Extracellular Polymeric Substance (EPS) Staining

**Figure 4:**
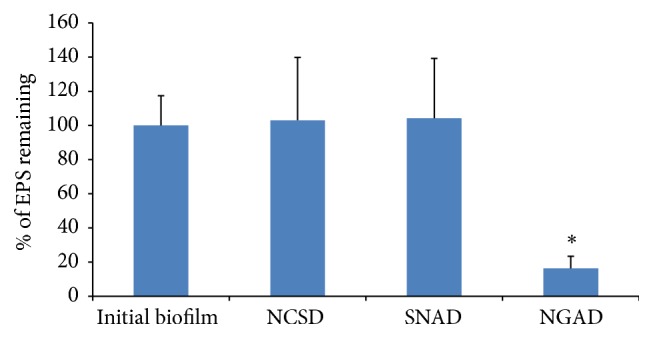
Percentage of EPS still remaining after exposure to the silver test dressings for 48 hours. ^*∗*^ *p* = 0.000.

**Figure 5:**
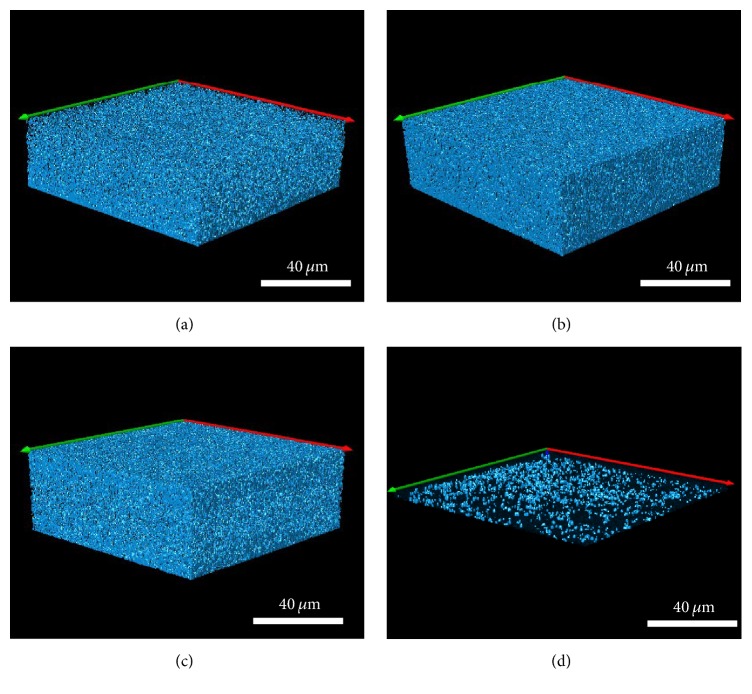
Representative isosurface 3D imaging, performed using the* Image-Pro Premier* 3D software, of the CLSM images biofilm EPS stained with Calcofluor White. (a) Initial biofilm *T*_24 hours_. (b) NCSD after 48 hours of exposure. (c) SNAD after 48 hours of exposure. (d) NGAD after 48 hours of exposure.

Calcofluor White stains the *β*-1,2 and *β*-1,3 polysaccharides present in the biofilm EPS [10]. The NGAD was the only dressing that was able to significantly reduce biofilm mass compared to the control biofilm (*p* = 0.000) (Figure 4) as indicated by this staining method. Neither NCSD (Figure 5(b)) nor SNAD (Figure 5(c)) dressings resulted in any bulk EPS reduction compared to the control biofilm (Figure 5(a)), while bulk EPS reduction by the NGAD was evident (Figure 5(d)).

#### 3.2.2. Total Viable Counts

**Figure 6:**
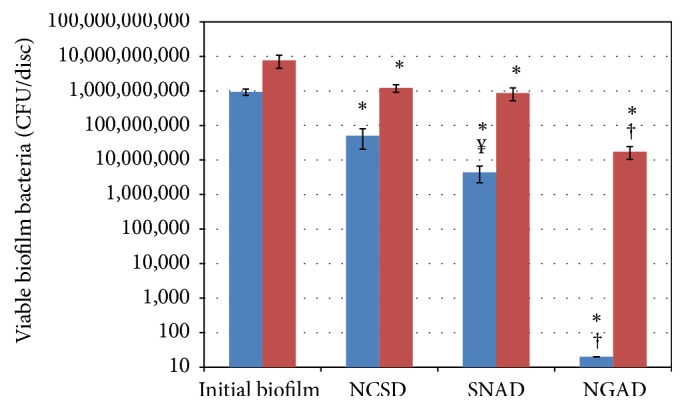
Total viable counts of (blue colour)* S. aureus* and (red colour)* K. pneumoniae* biofilm cells after 48 hours of exposure to silver dressings (*n* = 5). Initial biofilm = *T*_24 hours_. ^*∗*^ *p* < 0.05 compared to initial biofilm. ^†^ *p* = 0.000 compared to NCSD and SNAD. ^*¥*^ *p* = 0.027 compared to NCSD.

Total viable counts of* S. aureus* and* K. pneumoniae* biofilm cells after 48 hours of exposure to the silver test dressings are shown in Figure 6. Initial biofilms were predominantly comprised of* K. pneumoniae* cells despite its lower starting inoculum (see Materials and Methods), which may be attributable to the avid biofilm-forming capacity of this nosocomial pathogen [11]. In all instances,* K. pneumoniae *biofilm cells were more difficult to kill. Student's 2-sample *t*-tests showed that each silver dressing reduced viable biofilm cells of both species compared to the no-dressing control (NCSD: *p* = 0.017; SNAD: *p* = 0.015; NGAD: *p* = 0.000). Following ANOVA, simultaneous confidence interval comparisons demonstrated that there were statistically significant differences between silver dressings. The NGAD was significantly more effective than NCSD and SNAD at killing* S. aureus* biofilm cells (*p* = 0.000). The NGAD was also more effective than NCSD and SNAD at killing* K. pneumoniae* biofilm cells (*p* = 0.000). SNAD was significantly more effective than NCSD at killing* S. aureus* biofilm cells (*p* = 0.027), but there were no significant differences between the effects of NCSD and SNAD on* K. pneumoniae* biofilm cells (*p* = 0.098).

#### 3.2.3. PNA FISH

**Figure 7:**
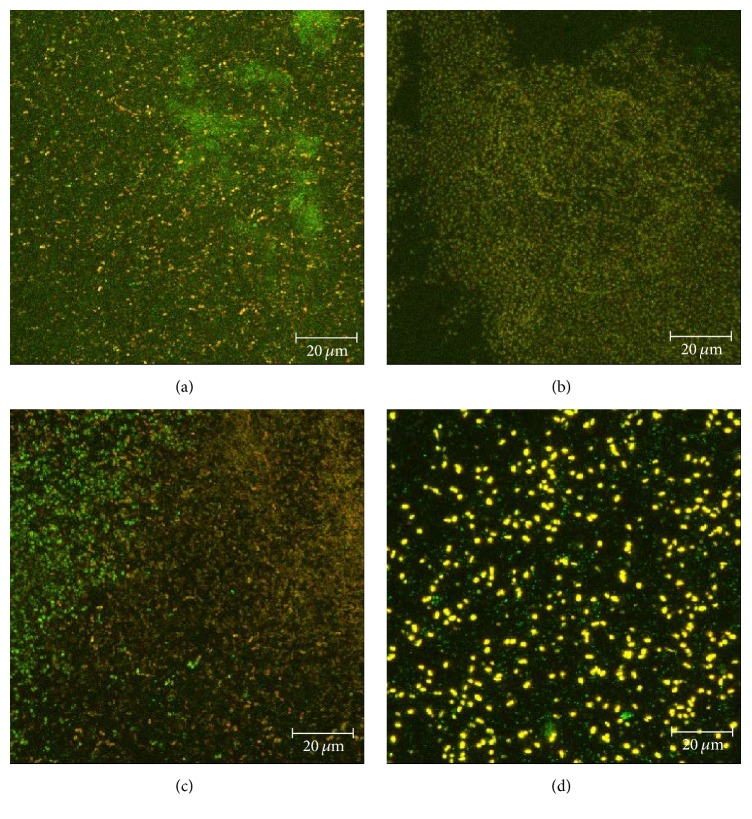
Representative CLSM images of polymicrobial biofilm where bacteria have been fluorescently tagged (green =* S. aureus*; yellow =* K. pneumoniae*). (a) Initial biofilm *T*_24 hours_; (b) NSCD after 48 hours; (c) SNAD after 48 hours; (d) NGAD after 48 hours.

The confocal PNA FISH images in Figure 7 generally correlate with the total viable count data (Figure 6). The representative images of the biofilms exposed to NCSD (Figure 7(b)) and SNAD (Figure 7(c)) showed a high frequency of both green and yellow objects, indicating high concentrations of* S. aureus *and* K. pneumoniae* cells, respectively. In contrast, the image for the NGAD shows fewer yellow objects, representative of the significantly lower* K. pneumoniae* counts, and feint green areas which are likely to be cell debris rather than viable* S. aureus* cells (Figure 7(d)). The individual yellow objects appear brighter because the amount of stain added to each sample was constant; therefore a greater amount of stain was available per cell present in the NGAD treated sample.

#### 3.2.4. Image Analysis

**Figure 8:**
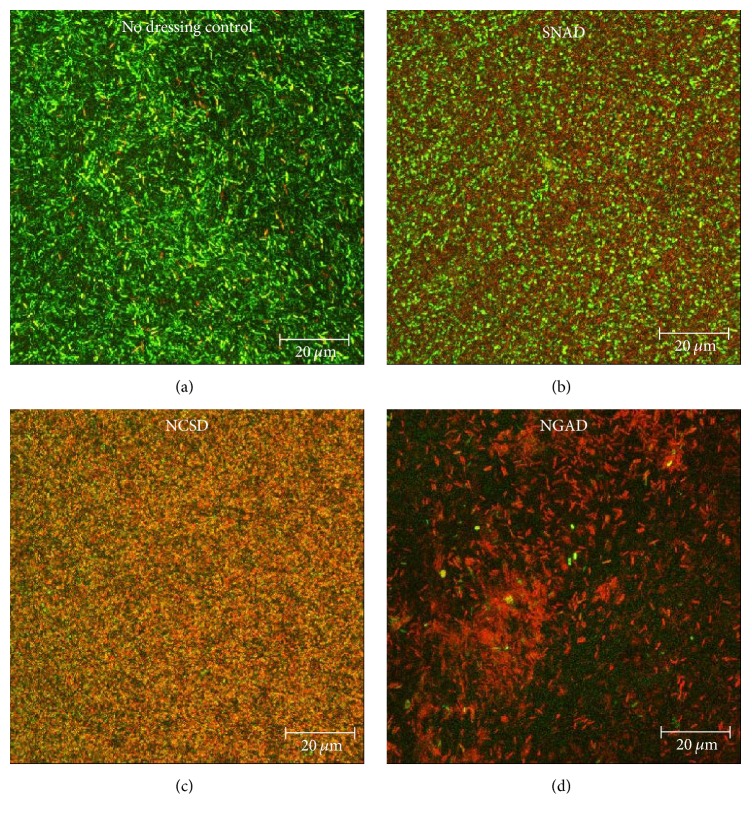
Composite (full thickness) images of antibiotic-resistant* P. aeruginosa* biofilm stained with* BacLight* after 48 hours of contact with the test dressings (green = viable bacteria; red = nonviable bacteria). (a) No-dressing control. (b) SNAD. (c) NCSD. (d) NGAD.

*Live/Dead BacLight* staining enables differentiation of live cells (green), mixed live and dead or dying cells (yellow/orange), and dead cells (red) in composite images as shown in Figure 8. These images are examples of sections found midway through the thickness of each sample. The control was predominantly living cells but there was some death due to natural turnover. As a general observation the apparent order of* in vitro* antimicrobial effectiveness of the dressings was NGAD > NCSD > SNAD.

**Figure 9:**
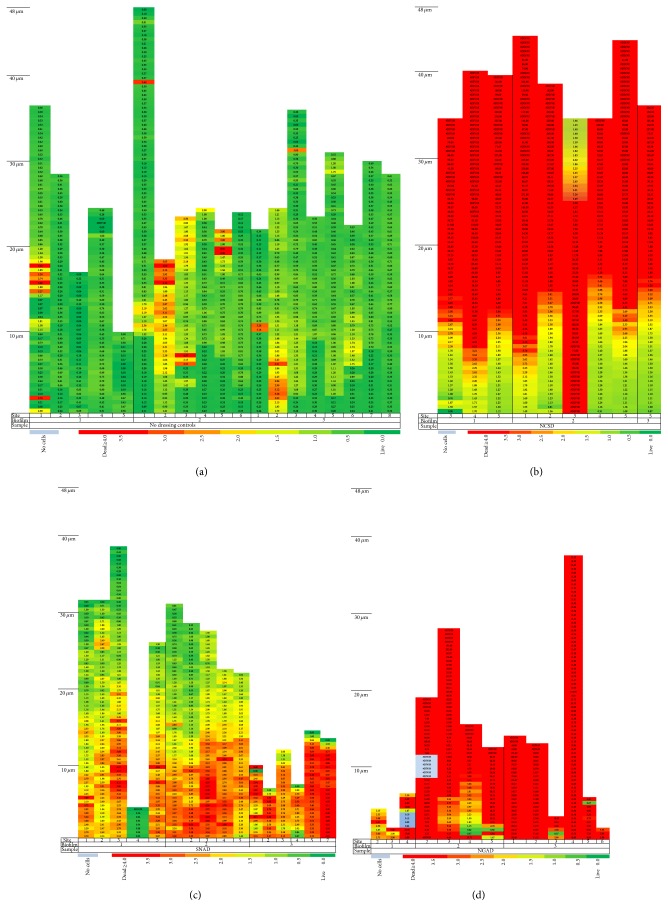
Colour-coded bacterial viability within biofilm layers as a function of distance from the filter surface for an antibiotic-resistant* P. aeruginosa* biofilm after 48 hours of contact with the test dressings (green = viable bacteria; red = nonviable bacteria). (a) No-dressing control. (b) NCSD. (c) SNAD. (d) NGAD.

However, as predicted in the 3D reconstructions in Figure 3, there were different and distinctive changes in the green-to-red ratio through the thickness of the biofilm.  Figure 9 is a colour-coded representation of how this ratio changes for the different silver dressings tested. The scale extends from green (where there were more than two green cells for every red cell) through yellow (where there were approximately two red cells for every green cell) to red (where there were at least four red cells for every green cell). Control biofilms were observed to be of varying thickness (between 10 and 48 *μ*m as indicated by the presence of stained cells) and predominantly viable (Figure 9(a)). Although NCSD showed a wide zone of bactericidal action at the point where the dressing contacted the biofilm, the thickness of the residual biofilm appeared unchanged and there was a biofilm survival zone approximately 10 *μ*m thick at the filter surface (Figure 9(b)). This indicates the inability of ionic silver alone to kill bacteria in deeper parts of the biofilm and may explain the recurrent nature of biofilm infections, as has also been observed with antibiotics [12]. Biofilm exposed to SNAD appeared to be of reduced thickness with zones of death near the filter surface, but there were areas of viable biofilm cells nearer the upper surface that would have been in contact with the dressing (Figure 9(c)). Many of the viewed sites for the NGAD appeared to have no residual biofilm (data not shown in Figure 9(d)), but where biofilm could be observed it was of much reduced thickness and was less integral in that it contained voids (regions in the depth of the sample that contained no cells), and in regions that did contain cells these were largely devoid of green cells (surviving bacteria) (Figure 9(d)). Green objects were observed in a few samples relatively close to the filter surface but this was the area in which image artefacts were most prevalent (e.g., deformations in the filter surface causing reflection).

#### 3.2.5. Elemental Composition of Biofilms

Divalent metal cations such as magnesium (Mg^2+^), calcium (Ca^2+^), and zinc (Zn^2+^) play an important role in the formation, adhesion, and cohesion of biofilm [13, 14] and are tightly bound into its structure. Therefore, measuring the sum of these divalent ions will give an indication of the relative amount and strength of biofilm and of the effect of the test dressings on biofilm disruption. Small monovalent cations such as sodium (Na^+^) and potassium (K^+^) are constant components in isotonic fluids and are not tightly bound to either biofilm, tissue, or dressings, so they can be used as an approximate measure of total mass present. The NGAD contains Na^+^ and SNAD contains Na^+^ and Ca^2+^ while NCSD contains neither; therefore comparison by following these ions was not possible. However, the elemental composition of CMC, SCMC, and the NGAD is very similar as they are all based on the same fibre; therefore these can be directly compared. Because Na^+^ was present in both biofilm and the test dressings, this could not be used to differentiate between residual biofilm and residual dressing; however K^+^ and divalent cations are absent from CMC, SCMC, the NGAD, and the filter disc; therefore any present would be indicative of biofilm only. The amount of silver (Ag^+^) detected and ratio to K^+^ and/or the total divalent metals (Metal^2+^ = the sum of Mg^2+^, Ca^2+^, and Zn^2+^) will be indicative of the effectiveness of antimicrobial action. The low concentration of these metal ions required the use of a trace elemental technique such as ICP-MS.

#### 3.2.6. Comparison of Carboxymethylcellulose-Based Fibre Dressings

The entire biofilm remaining on the filter disc after 24 hours of contact with hydrated dressings was analysed using a standardised sample preparation method. The comparative amount of each analyte determined is shown in Table 5. The absolute amount of K^+^ and Metal^2+^ in the biofilm control and the amount of Ag^+^ in SCMC are designated as 100%.

With the exception of K^+^ for* C. albicans *biofilm, the trends in assays of K^+^ and Metal^2+^ were in general agreement showing that the base CMC dressing had the ability to significantly reduce biofilm mass in each biofilm type (*p* < 0.05; mean relative reduction across the biofilm types compared to the no-dressing control of 67% and 69% for K^+^ and Metal^2+^, resp.). A similar biofilm mass reduction was also observed for SCMC (average for all types of biofilm of 75% (K^+^) and 68% (Metal^2+^)) with a statistically significant increased reduction in CA-MRSA biofilm for SCMC compared to CMC. The NGAD formulation further increased the mean reduction in K^+^ and Metal^2+^ (to 82% and 76%, resp.), with the NGAD being significantly more effective than SCMC at reducing both biofilm markers in* P. aeruginosa* biofilm (K^+^ *p* = 0.035; Metal^2+^ *p* = 0.014) and Metal^2+^ in* C. albicans* biofilm (*p* = 0.010).

The NGAD dressing induced a statistically significant greater silver uptake in all biofilm types compared to the SCMC dressing (which contained the same amount, type, and form of silver), on average 134% more. Although the* C. albicans* biofilm appeared to be the most difficult biofilm to manage, being reduced in mass the least (~70% by the NGAD), the amount of silver within the residual biofilm was approximately 42% higher for the NGAD compared to the SCMC dressing (*p* = 0.014). For the* P. aeruginosa *biofilm the silver content was 81% higher after management with the NGAD than SCMC (*p* = 0.006) and the CA-MRSA biofilm seemed the most susceptible to the effects of the NGAD with approximately three times more silver (278%; *p* = 0.000) than SCMC.

#### 3.2.7. Comparison of Different Forms of Silver-Containing Dressings

Due to compositional interferences it was not possible to chemically compare the effects of different silver-containing dressing types on the weakening and removal of biofilm mass. However, it was possible to directly compare the donation of silver into residual antibiotic-resistant* P. aeruginosa* biofilm after 48 hours of exposure to the test dressings. These same samples were also subjected to live/dead staining and image analysis which enabled biofilm thickness to be estimated based on the presence of bacterial cells in image stacks.  Table 6 summarises this data as averages (statistical analysis has been performed for individual assays), and it also calculates the mass of silver per unit thickness of the residual biofilm, comparing this to the silver per unit area of dressing initially applied.

The residual biofilm after management with the NGAD contained 5-times the absolute amount of silver and more than 16-times the concentration of silver per unit biofilm thickness compared to that observed for NCSD managed biofilm (*p* = 0.000); this was despite the NGAD only containing approximately one-ninth of the silver in NCSD on a weight per dressing area basis. The residual biofilm managed with the NGAD contained 17-times the amount of silver (*p* = 0.000) and more than 30-times the concentration of silver per unit biofilm thickness compared to SNAD (*p* = 0.000), with the NGAD containing less than one-sixth of the amount of silver on a weight per area basis. Residual biofilm after management with NCSD contained significantly more silver than SNAD on an absolute amount basis (*p* = 0.009) but not on a concentration per thickness basis (*p* = 0.063).

## 4. Discussion

Chronic wounds are invariably associated with poor healing and susceptibility to recurrent infections, and this is characteristic of a biofilm-induced chronic condition. Consequently, in order to minimise the opportunity for wound infection and encourage healing, there is a need to manage biofilm effectively. Uses of standard antibiotics and antiseptics are not necessarily the immediate solution because biofilm is notoriously tolerant to these antimicrobial agents [15]. Consequently new strategies are required to eliminate biofilm and expose associated microorganisms to make them more vulnerable to antimicrobial agents. While wound bed preparation, involving debridement and cleansing, is an ideal way to physically reduce bioburden and help expose microorganisms before dressing the wound [16, 17], it is unlikely to entirely eradicate biofilm and debridement methodologies and effective wound cleansers are not always available to all wound care practitioners in all settings. The most efficient way to provide longer-term antimicrobial action to a wound is therefore via antimicrobial dressings, and the recognition of biofilm as a key barrier to wound healing within the last decade [4, 5] has provided a new challenge to developers of therapeutic dressings.

**Figure 10:**
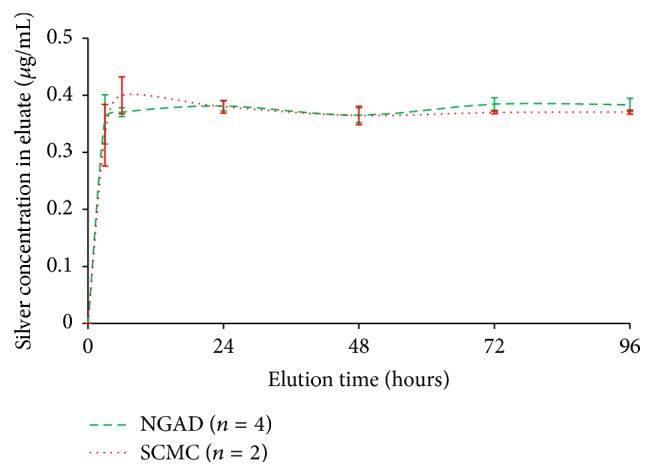
Comparison of elution of silver ions from the NGAD and SCMC into a constantly stirred excess of isotonic media (0.9% w/v NaCl_(aq)_, 8 mL per cm^2^ dressing at 37 ± 3°C) as determined by ICP-MS.

The NGAD described in this* in vitro* study is a proprietary, highly innovative wound dressing designed to help the antimicrobial silver component work most effectively by disrupting the protective components of biofilm. Namely, this involved the careful selection of a synergistic combination of safe antibiofilm excipients [9], 0.39% disodium ethylenediaminetetraacetate (a metal chelator), 0.135% benzethonium chloride (a surfactant), and close pH control (5.0 to 6.0), to add to the formulation of a widely used silver Hydrofiber dressing (SCMC) [2]. The SCMC dressing was formulated prior to 2002, before the realisation of the significance of biofilm in wound care. Its purpose was to assist in the prevention and management of infection; therefore it was targeted at planktonic bacteria against which it is proven to be highly effective* in vitro* [1]. The NGAD has the same base formulation as SCMC (sodium silver CMC fibres containing 1.2% silver ions) and has been shown to have the same physical performance* in vitro* [7, 9], biocompatibility, and an equivalent clinical safety profile [18, 19]. In elution studies into isotonic media the silver ion release profiles of the NGAD and SCMC have also been shown to be equivalent (Figure 10). NGAD dressings prepared without silver have been shown to have no antimicrobial activity in standard log-reduction models against planktonic pathogenic wound bacteria* in vitro* (data not shown).

The* in vitro *study described here was designed to assess the antibiofilm and antimicrobial characteristics of the NGAD compared with other silver-containing dressings and further elucidate its mode(s) of action. In a program of increasingly complex and challenging biofilm models, this study compared the antibiofilm activity of this next generation dressing to existing silver dressings. The dressing characteristics examined were the following.

*(1) The Ability of the Dressings to Disrupt Biofilm*. The biofilm-disrupting effect of the NGAD formulation appears to act synergistically with the inherent biofilm removal capability of Hydrofiber technology. This was demonstrated by elemental analysis in reductions in biofilm-associated ions (K^+^ and Metal^2+^), and the NGAD resulted in some significantly greater (*p* < 0.05) biofilm-disruptive effects than the base CMC and the silver-containing SCMC, depending on biofilm type. The Hydrofiber technology used in the NGAD has previously been shown to sequester cells* in vitro *[20], and it is therefore likely that EPS loosened or broken up by the additional components of the NGAD were also sequestered into the dressing, as supported by the EPS reduction via Calcofluor White staining. 

*(2) The Ability of the Dressings to Absorb Biofilm and Reduce Biomass*. In addition to the EPS reduction effected by the NGAD, the reduction in the number of biofilm cells and biofilm thickness was demonstrated by live/dead staining and colorimetric image analysis, further supporting the synergy between the antibiofilm action of the formulation and the physical sequestration capability of Hydrofiber technology.

*(3) Ability of the Dressings to Donate Antimicrobial Silver to Biofilm Cells*. Elemental analysis showed that the NGAD donated significantly more (*p* < 0.05) silver ions to biofilm than the standard silver-containing Hydrofiber dressing and the other commercial silver-containing dressings tested (despite this latter group of dressings containing more silver). It is apparent that simply adding more silver to wound dressings is unlikely to be the most effective way of killing biofilm microorganisms. The elution rate of ionic silver into isotonic media is the same for SCMC and the NGAD, so the enhanced silver donation by the NGAD can only be attributed to the antibiofilm formulation, disodium ethylenediaminetetraacetate, benzethonium chloride, and pH control, removing the EPS barrier and enhancing the efficiency of transfer of the antimicrobial agent into the biofilm cells. 

*(4) The Ability of the Dressings to Kill Biofilm-Associated Microorganisms*. As may be expected due to the enhanced biofilm penetration of silver ions, the biofilm viable count data was aligned with colorimetric image analysis and live/dead staining. Despite the NGAD containing notably lower concentrations of ionic silver, the NGAD significantly outperformed (*p* = 0.000) the other silver dressings in killing biofilm cells in a challenging polymicrobial biofilm model.

**Figure 11:**
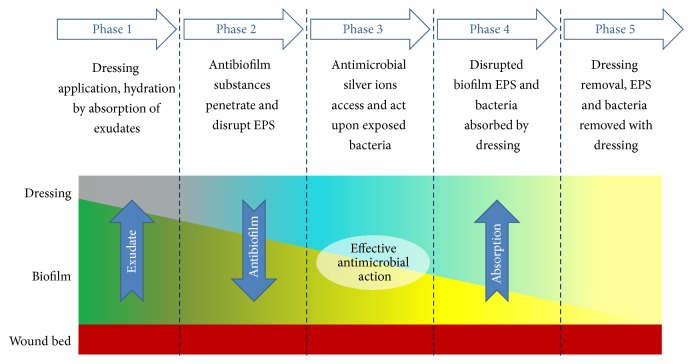
Functionality of the NGAD.

Irrespective of the microscopic, analytical, or microbiological method used to analyse the antibiofilm effects, the NGAD was shown to reduce biofilm thickness and reduce biofilm cell viability compared to standard silver wound dressings, despite these containing notably higher silver concentrations. This observation supports recently reported clinical observations, where static or deteriorating chronic wounds that had been unsuccessfully managed with, amongst others, standard silver dressings were dramatically improved following a switch to the NGAD in otherwise standard care protocols [18]. The enhanced antibiofilm and antimicrobial action observed in this study helps to explain the encouraging early* in vitro* [7, 9, 21],* in vivo* [22], and clinical results [18, 19, 23] observed for this next generation dressing technology and sheds further light on its modes of action. Based on the* in vitro* data generated in this study, the functionality of the NGAD can be described in five phases (Figure 11).

**Phase 1 .**

The applied NGAD dressing hydrates and gels on contact with wound fluids, contacting intimately [2] the wound bed and surface biofilm.

**Phase 2 .**

Biofilm is loosened and dispersed due to the synergistic action of the disodium ethylenediaminetetraacetate and benzethonium chloride in combination with sodium silver CMC fibres [9].

**Phase 3 .**

Exposed microorganisms become highly susceptible to killing by the action of ionic silver.

**Phase 4 .**

Residual biofilm and cells are immobilised within the gelled dressing.

**Phase 5 .**

Biofilm biomass is reduced by dressing removal.

## 5. Conclusions

This* in vitro *study offers new insight into the antimicrobial and antibiofilm behaviour of dressings against clinically relevant microbial forms (biofilm) and how those microorganisms respond to dressing technology. An antimicrobial dressing technology (formulation and physical properties) influences its ability to expose bacteria to the antimicrobial agent. The NGAD, with its specifically designed biofilm-disrupting formulation, ionic silver and Hydrofiber base was the most effective dressing at disrupting, killing, and removing biofilm and donating the greatest amount of silver into the residual biofilm, despite the dressing containing the least silver of the dressings tested. Antimicrobial efficacy against biofilm cannot be predicted by silver type or form, silver content, or silver elution data. This* in vitro* study improves our understanding of how this new dressing technology is effective, both in the laboratory and in the clinic.

## Figures and Tables

**Table 1:** Testing matrix.

Challenge microorganism	Biofilm growth medium (BGM)	Model	Test methodology	Dressings tested (Table 2)
Antibiotic-resistant* Pseudomonas aeruginosa* NCTC 13437 Community-acquired Methicillin-resistant *Staphylococcus aureus* (CA-MRSA) USA300 *Candida albicans *NCPF 3179	Foetal calf serum (FCS)	Simple biofilm model	Elemental analysis (K^+^, Mg^2+^, Ca^2+^, Zn^2+^, Ag^+^) of residual biofilm after dressing being applied for 24 hours (a simple and chemically consistent model to investigate formulation effects) (*n* = 6)	CMC SCMC NGAD
Antibiotic-resistant *P. aeruginosa* NCTC 8506			Elemental analysis (Ag^+^) and quantitative live/dead staining after dressing being applied for 48 hours (a more challenging strain and longer dressing exposure time selected to challenge the silver-containing dressings) (*n* = 9)	NGAD NCSD SNAD

*S. aureus* (clinical wound isolate, CI72) and* Klebsiella pneumoniae *(clinical isolate, CI45)	TSB : FCS (50 : 50)	Simulated wound polymicrobial biofilm model	Quantitative microbiology (total viable counts), PNA FISH, live/dead and Calcofluor White staining after dressing application for 48 hours (a more complex inoculum and longer dressing exposure time selected to challenge the silver dressings) (*n* = 5)	NGAD NCSD SNAD

**Table 2:** Test dressings. ^†^Formulation proprietary to ConvaTec Ltd.

Commercial name	Physical and chemical composition	Coding
AQUACEL Extra	Two layers of a needle-punched nonwoven fleece of sodium carboxymethylcellulose (CMC) fibres	CMC
AQUACEL Ag Extra	Two layers of a needle-punched nonwoven fleece of sodium silver CMC fibres (approximately 1.2% w/w or 0.17 mg/cm^2^ silver) [2]	SCMC
AQUACEL Ag+ Extra	Two layers of a needle-punched nonwoven fleece of sodium silver CMC fibres enhanced with disodium ethylenediaminetetraacetate and benzethonium chloride^†^, stitched with a high purity cellulose thread (approximately 1.2% w/w or 0.17 mg/cm^2^ silver)	NGAD
Acticoat™ 7	Three layers of a metallic (nano) crystalline silver-encrusted high-density polyethylene (HDPE) mesh alternating with two layers of a rayon polyester nonwoven fabric, bonded at intervals by ultrasonic welding of the HDPE (approximately 8.4% w/w or 1.48 mg/cm^2^ silver)	NCSD
Silvercel™ Non-Adherent	A nonwoven fabric comprised of a blend of metallic silver-coated nylon fibres and calcium alginate/CMC fibres between two apertured sheets of ethylene methyl acrylate (EMA) (approximately 4% w/w or 1.11 mg/cm^2^ silver [8])	SNAD

**Table 3:** Reproducibility of the biofilm models utilised, as analysed by different analytical, imaging, and microbiological assays.

Model	Strain	Analysis method	*n*	Average	Std Dev
Simple biofilm model	Antibiotic-resistant *P. aeruginosa* NCTC 8506	Elemental analysis (values minus blank filter disc elemental analysis)	24	26.8 *µ*M Mg^2+^	3.2 *µ*M
				36.2 *µ*M K^+^	7.1 *µ*M
				171.9 *µ*M Ca^2+^	27.0 *µ*M
	Antibiotic-resistant *P. aeruginosa* NCTC 13437	Live/dead image analysis by pixilation	19	25.6 *µ*m thick	8.2 *µ*m

Simulated wound polymicrobial biofilm model	*S. aureus*, *K. pneumoniae*, 24-hour control	Confocal imaging of maximum depth	15	9.74 *µ*m thick	2.37 *µ*m
		Total viable counts	3	9.4 × 10^8^ cfu *S. aureus*	2.0 × 10^8^ cfu
				7.7 × 10^9^ cfu *K. pneumoniae*	3.2 × 10^9^ cfu

**Table 4:** Depths (*µ*m) of polymicrobial biofilm (as indicated by the presence of bacterial cells) after exposure to silver dressings for 48 hours. Dressings were tested in triplicate and five images were captured for each dressing (*n* = 15). ^*∗*^ *p* < 0.000 compared to initial biofilm. ^†^ *p* < 0.000 compared to NCSD and SNAD.

Sample	Maximum depth (*µ*m) [mean ± standard deviation]
Initial biofilm *T*_24 hours_	9.75 ± 2.37
NCSD	6.22 ± 5.93^*∗*^
SNAD	4.77 ± 1.91^*∗*^
NGAD	1.99 ± 1.22^*∗*†^

**Table 5:** Metal assay results, average of *n* = 6. Statistical comparisons of NGAD to ^*∗*^ *p* = 0.000 compared to no-dressing control. ^a^ *p* = 0.018 compared to no-dressing control. ^b^ *p* = 0.007 compared to no-dressing control. ^c^ *p* = 0.016 compared to CMC. ^d^ *p* = 0.035 compared to SCMC. ^e^ *p* = 0.006 compared to SCMC. ^f^ *p* = 0.001 compared to CMC. ^g^ *p* = 0.014 compared to SCMC. ^h^ *p* = 0.013 compared to no-dressing control. ^i^ *p* = 0.009 compared to no-dressing control. ^j^ *p* = 0.010 compared to no-dressing control. ^k^ *p* = 0.027 compared to CMC. ^l^ *p* = 0.004 compared to CMC. ^m^ *p* = 0.038 compared to CMC. ^n^ *p* = 0.000 compared to SCMC. ^o^ *p* = 0.017 compared to no-dressing control. ^q^ *p* = 0.015 compared to no-dressing control. ^r^ *p* = 0.010 compared to SCMC. ^s^ *p* = 0.014 compared to SCMC.

Sample	*P. aeruginosa*			CA-MRSA			*C. albicans*		
	K^+^	Metal^2+^	Ag^+^	K^+^	Metal^2+^	Ag^+^	K^+^	Metal^2+^	Ag^+^
No-dressing control	100%	100%	0%	100%	100%	1%	100%	100%	0%
CMC	32%^a^	36%^*∗*^	0%	17%^h^	20%^*∗*^	0%	50%	37%^*∗*^	0%
SCMC	33%^a^	35%^*∗*^	100%	9%^il^	17%^*∗*^	100%	34%^o^	44%^*∗*^	100%
NGAD	12%^bcd^	24%^*∗*fg^	181%^e^	10%^jk^	15%^*∗*m^	378%^n^	31%^q^	33%^*∗*r^	142%^s^

**Table 6:** Biofilm thickness, amounts, and concentrations of silver in residual biofilm after 48 hours of dressing exposure and concentration of silver in the original applied dressings. *n* = 6 for silver assays and *n* = 9 or greater for mean biofilm thickness data. ^*∗*^ *p* = 0.000 compared to SCSD and SNAD. ^†^ *p* = 0.009 compared to SNAD.

	No dressing	NCSD	SNAD	NGAD
Mean biofilm thickness (*µ*m)	25.6	37.8	20.0	12.1
Amount of silver in residual biofilm (*µ*g)	0.0	14.1^†^	4.5	79.1^*∗*^
Concentration of silver in residual biofilm (*µ*g/*µ*m)	0.0	0.4	0.2	6.5^*∗*^
Concentration of silver in applied dressing (mg/cm^2^)	0.0	1.48	1.11	0.17
